# Supplementary material for: Multi-protein assemblies orchestrate co-translational enzymatic processing on the human ribosome
Source: Nat Commun. 2024 Sep 3;15:7681. doi: 10.1038/s41467-024-51964-9 (PMC11372111; doi:10.1038/s41467-024-51964-9)
Supplement: Supplementary file 1 — Supplementary Information [file 41467_2024_51964_MOESM1_ESM.pdf]

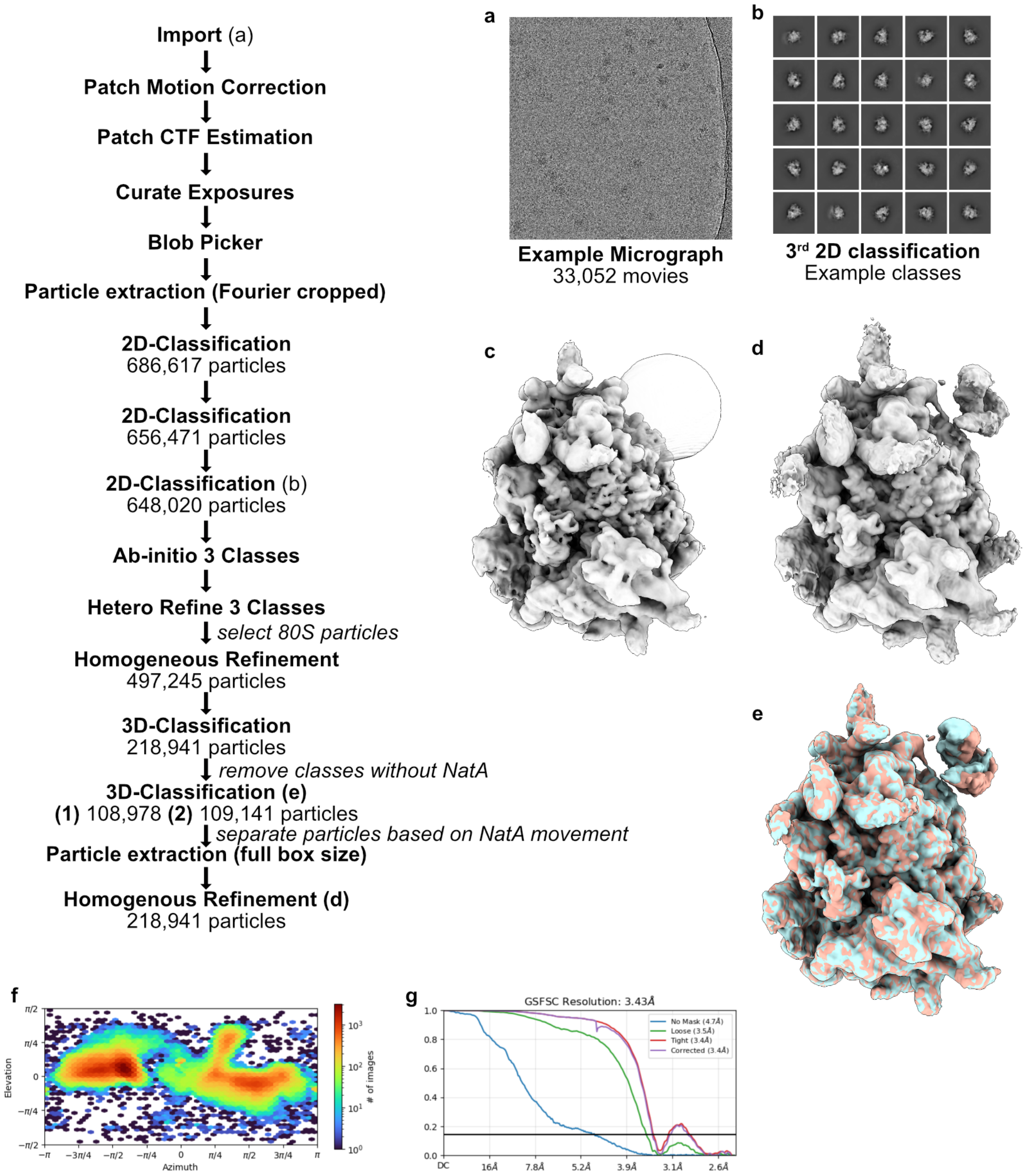

Supplementary Figure 1 | Cryo-EM data processing for the human NatA-80S sample. (a) Two datasets were acquired to obtain 33,052 movies. **(b)** After pre-processing in CryoSPARC, extracted particles were subjected to three rounds of 2D-classification. Three ab-initio classes were generated from the remaining particles and used to seed a Heterogeneous refinement. 80S particles were selected and subjected to Homogeneous Refinement. **(c)** A spherical mask was generated to encompass the distal site of NatA and used to initialize a 3D classification to remove particles without NatA. **(d)** Particles with NatA were subjected to Homogeneous refinement. **(e)** To further subclassify particles based on the motion of NatA, a second 3D classification into two classes was performed. Both particle subsets were finally subjected to Homogeneous refinement and superimposed to visualize the differences in NatA binding. **(f)** Angular distribution plot of particles used in the Homogeneous refinement shown in (d). **(g)** FSC curves from the Homogeneous refinement shown in (d).

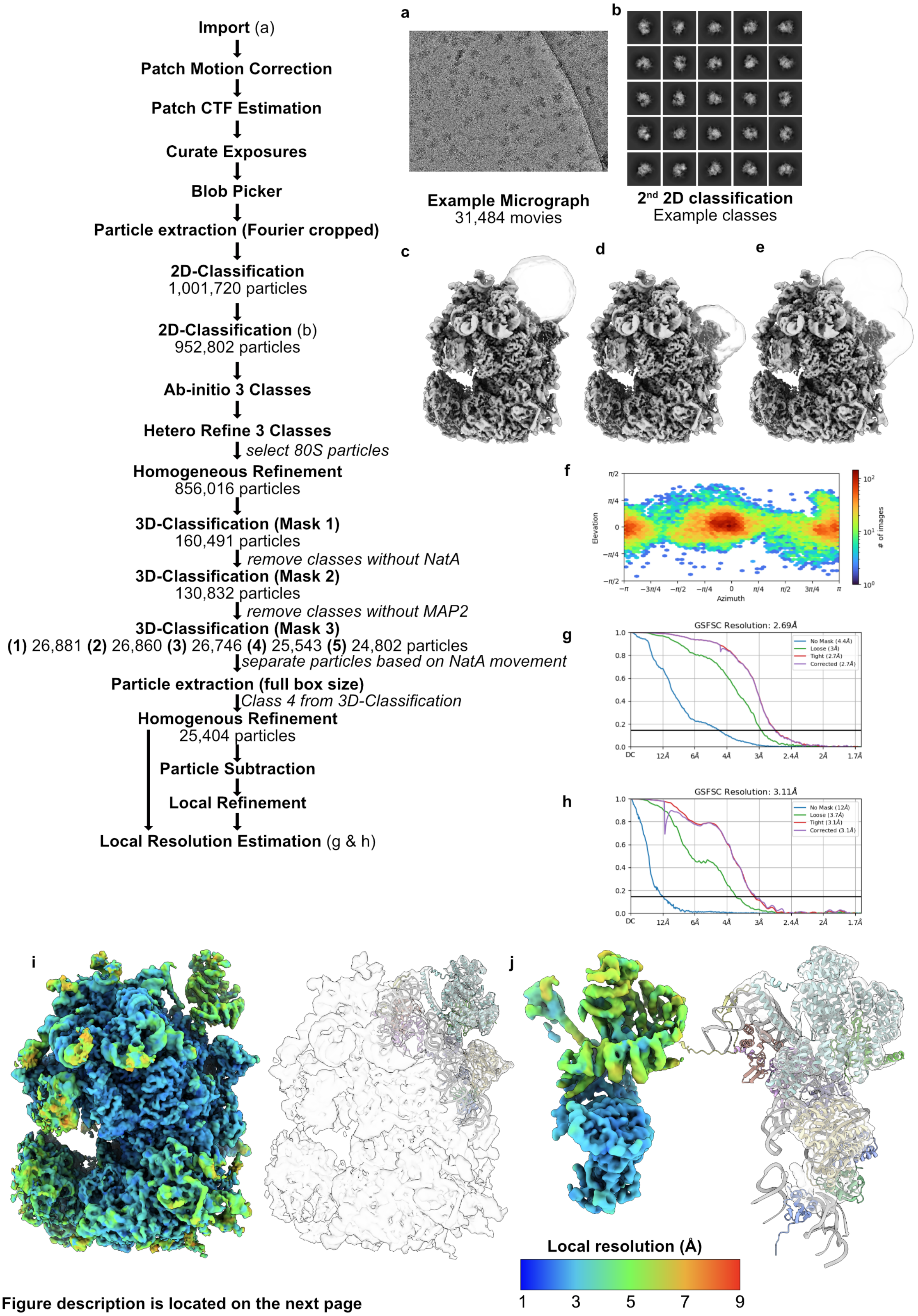

Figure description is located on the next page

Supplementary Figure 2 | Cryo-EM data processing for the human NatA-MAP2-80S sample. (a) A dataset was acquired to obtain 31,484 movies. **(b)** After pre-processing in CryoSPARC, extracted particles were subjected to two rounds of 2D-classification. Three ab-initio classes were generated from the remaining particles and used to seed a Heterogeneous refinement. 80S particles were selected and subjected to Homogeneous Refinement. **(c)** A spherical mask was generated to encompass the distal site of NatA and used to initialize a 3D classification to remove particles without NatA. **(d)** A second mask was made to encompass the binding site of MAP2 and used to initialize a second 3D classification to remove particles without MAP2. **(e)** A larger mask was generated to encompass the binding site of NatA and MAP2 and used for a third 3D classification to sort particles based on the motion of NatA. One class yielded a higher local resolution around NatA. Corresponding particles were re-extracted without Fourier cropping and subjected to Homogeneous refinement. **(f)** Angular distribution of particles used for the final Homogeneous refinement. **(g)** FSC plots of the final Homogeneous refinement. **(h)** Finally, as mask was generated to subtract the 80S signal and the mask shown in (e) was used to initialize a local refinement around MAP2 and NatA. The corresponding FSC curve is shown. **(h)** Local resolution estimation of the final Homogeneous refinement. **(i)** Local resolution estimation of the final local refinement. The final models are shown next to the local resolution images within the transparent map.

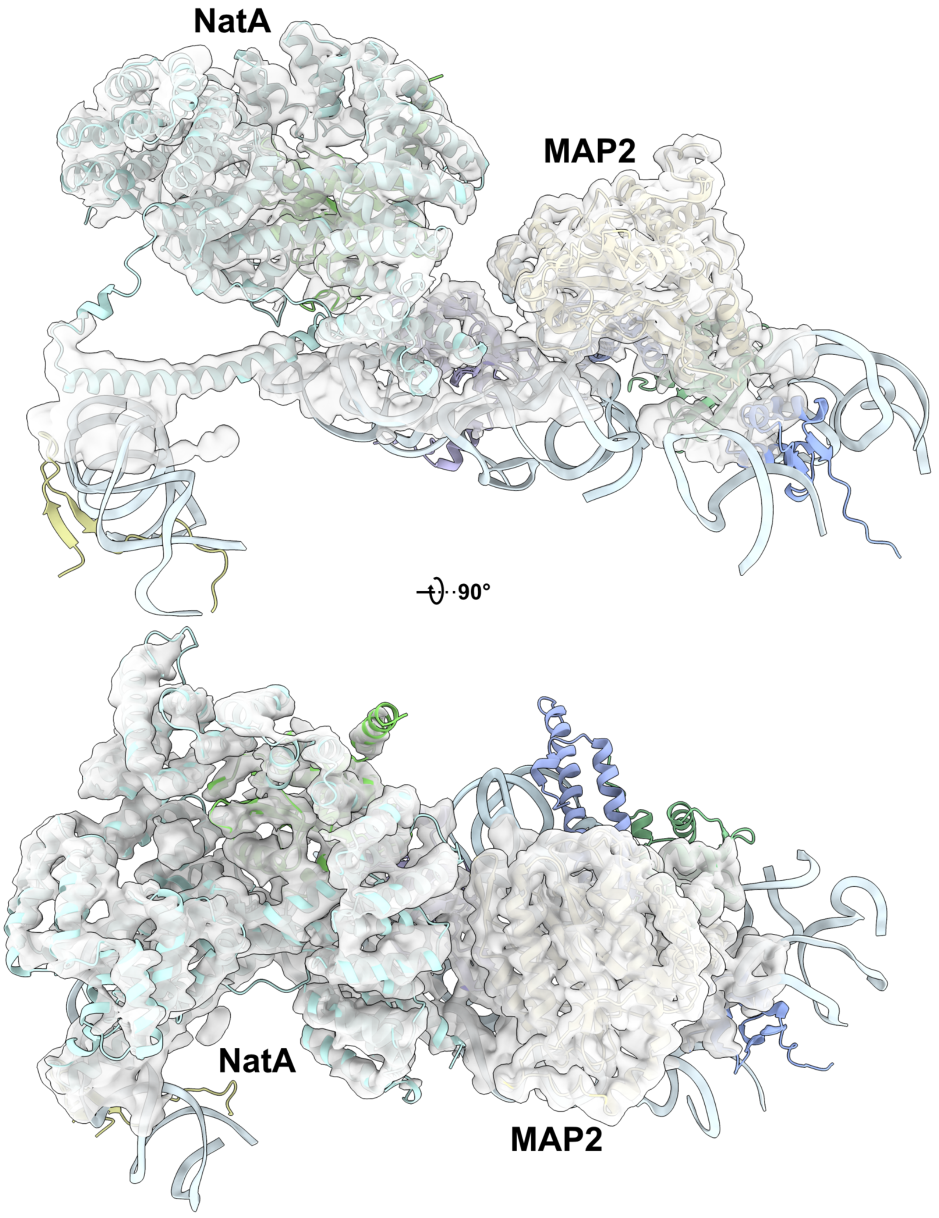

Supplementary Figure 3 | Structure of NatA-MAP2 on the human ribosome. Side and top view are shown onto NatA and MAP2 at the human ribosome. Both enzymes are shown as in Figure 1a. The cryo-EM map is shown in transparent. The local resolution around MAP2 is <3 Å while the local resolution around NatA is between 4 and 7 Å, as shown in Supplementary Figure 2.

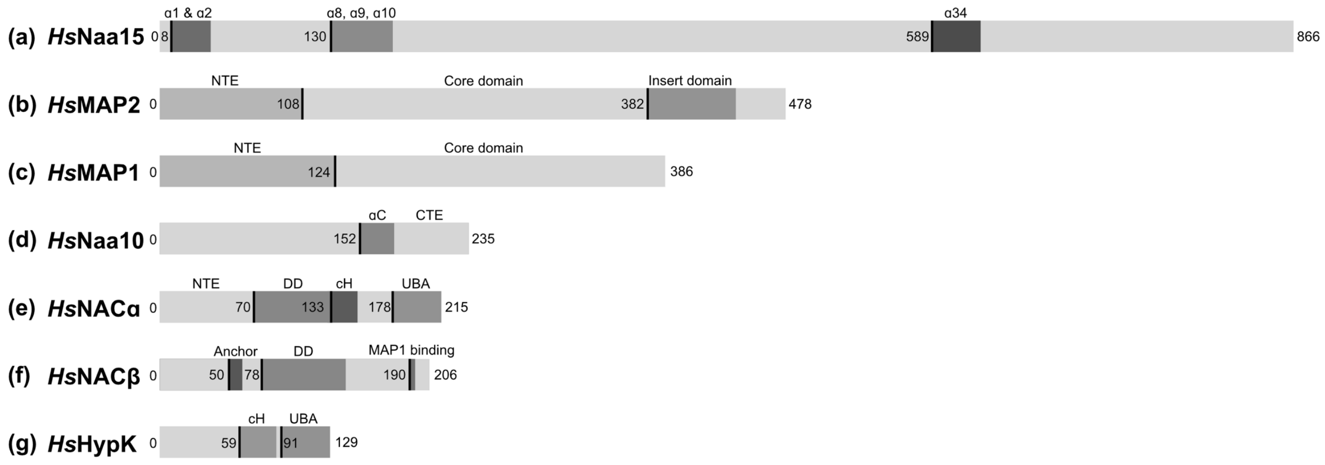

Supplementary Figure 4 | Domain organization of ribosome associated factors. (a) Domain organization of Naa15. The ribosomal contacts (helices α1, α2 and α34) are highlighted, as well as the helices α8, α9 and α10 which contact the NACα-cH and HypK-cH. **(b)** Domain organization of MAP2. The length of the unstructured N-terminal extension, as well as the position of the insert domain are indicated. **(c)** Domain organization of MAP1. The N-terminal extension (NTE) and core domain are highlighted. **(d)** Domain organization of Naa10. The C-terminal helix (αC) and unstructured C-terminal extension are labelled. **(e)** Domain organization of NACα. The dimerization domain (DD) is located centrally within the protein sequence. Adjacent to the dimerization domain, the NACα-cH that contacts Naa15 is highlighted, as well as the three-helix bundle UBA domain. **(f)** Domain organization of NACβ. The dimerization domain (DD) is located centrally within the protein sequence. N-terminally, the helical anchor which mediates ribosome binding is highlighted. C-terminally, the conserved hydrophobic stretch that mediates MAP1 recruitment is highlighted[1]. **(g)** Domain organization of the NACα homologue HypK. The HypK-cH that contacts Naa15 is highlighted, as well as the UBA domain.

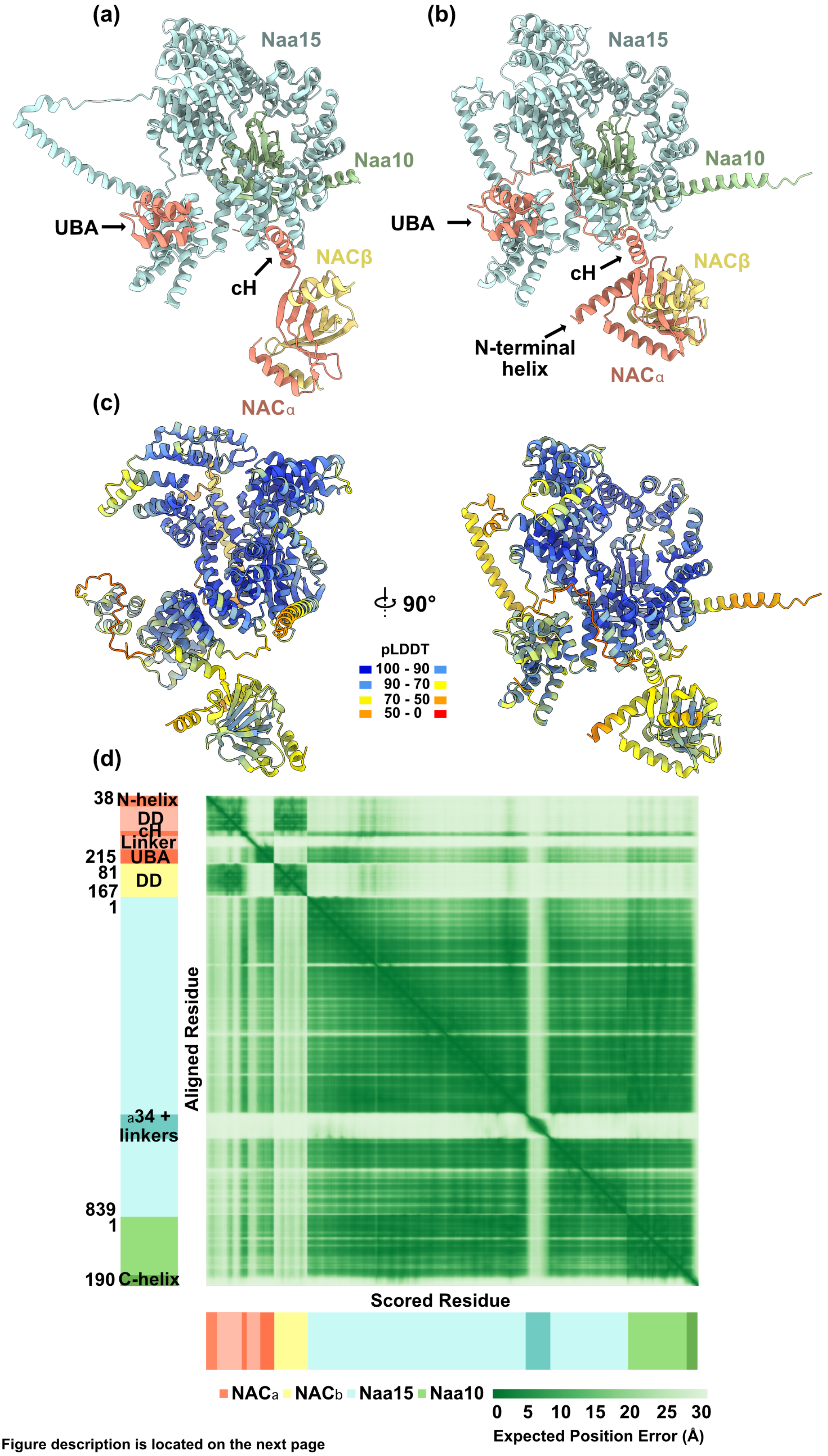

Figure description is located on the next page

Supplementary Figure 5 | Alphafold 3 prediction of the NAC-NatA interaction compared to the experimental cryo-EM structure at the ribosome. (a) The cryo-EM structure of the quaternary NatA-NAC-MAP1-80S assembly reveals contacts between NACα and Naa15 mediated by the NACα-cH and the NACα-UBA domain. **(b)** Alphafold 3 prediction[2] of the NatA-NAC interaction. The prediction accurately places the NACα-cH and UBA domain on Naa15, agreeing with the position and orientation of the experimental structure. Part of the N-terminal NACα extension is predicted to form a helix. **(c)** Two viewing angles on the Alphafold 3 prediction, coloured by the pLDDT score. **(d)** Confidence plot of the Alphafold 3 prediction. To the left and below the confidence plot, the corresponding proteins are labelled and regions of interest are highlighted (NACα N-terminal helix, NACα dimerization domain (DD), NACα-cH, the linker that connects the NACα-cH to the NACα-UBA domain, the NACα-UBA domain, the NACβ-DD, the Naa15-α34 anchor and linker, as well as the C-terminal Naa10 helix). To improve the readability of the confidence plot, unstructured regions that are not involved in the NACα-NatA interaction were removed. The residues that were used for the prediction are shown on the left (NACα:38-215, NACβ:81-167, Naa15:1-839, Naa10:1-190). The NACα-cH and UBA domain are confidently predicted relative to Naa15.

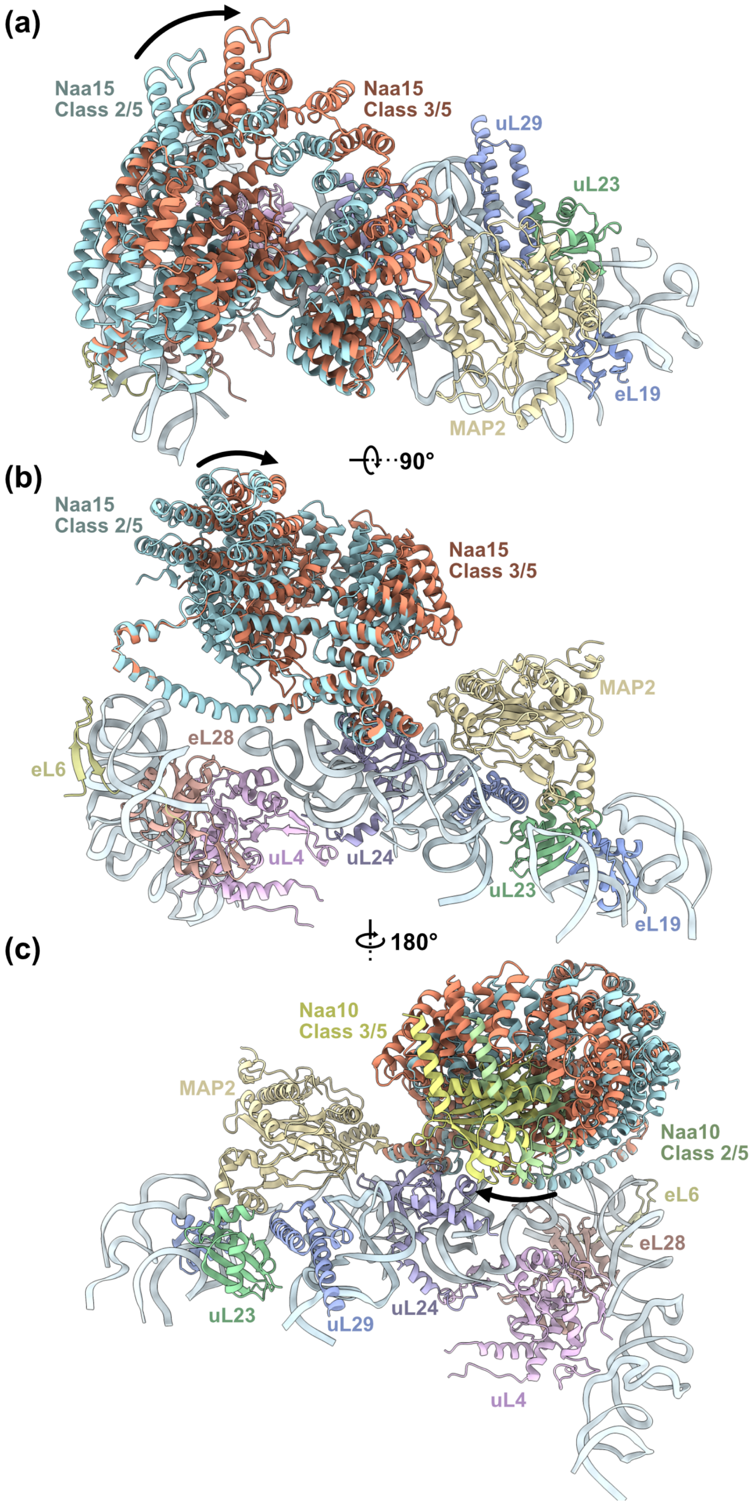

Supplementary Figure 6 | Dynamics of NatA in the ternary NatA-MAP2-80S complex. 3D classification revealed that NatA still moves in the distal site, despite the presence of MAP2. In total, five classes were obtained in the final 3D classification with slightly different modes of NatA binding **(Supplementary Fig. 2)**. Classes 2 and 3 showed the strongest differences and are compared in this figure. **(a)** NatA twists and **(b)** rotates in the distal site but retains all contacts to the ribosome. **(c)** When NatA rotates towards the PTE, Naa10 is shifted along the surface of uL24.

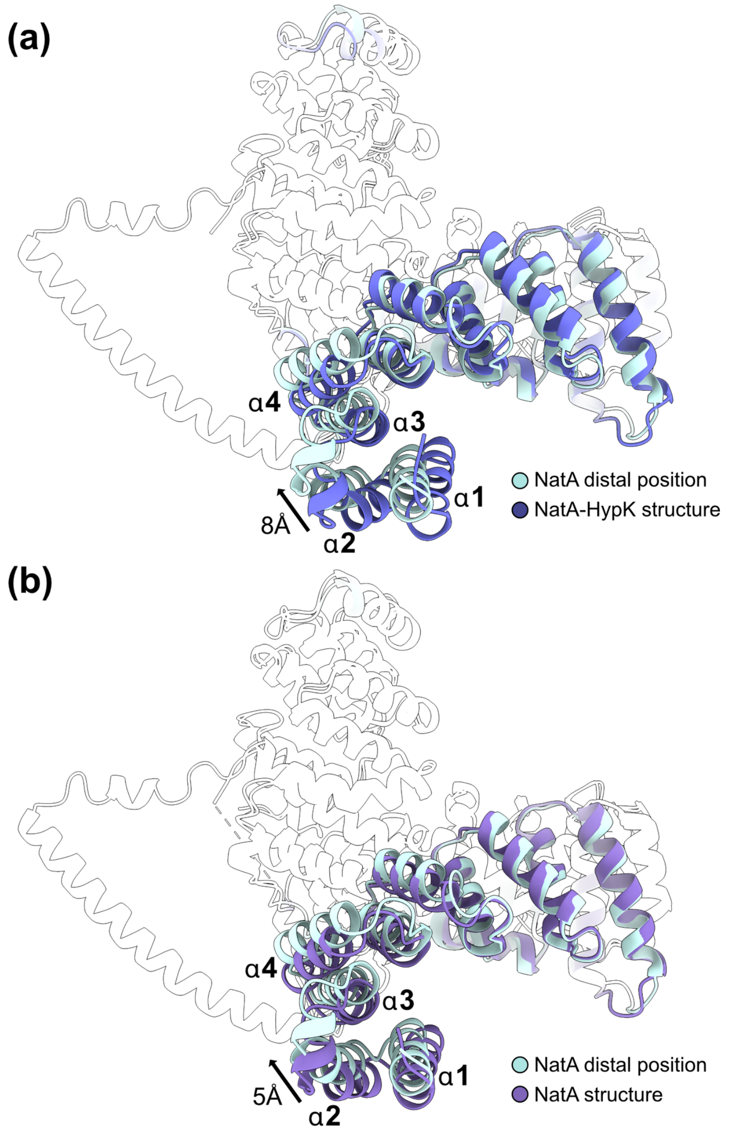

Supplementary Figure 7 | Adaptation of the Naa15 TPR-scaffold to fit the distal position on the ribosome. (a) Compared to the structure of NatA alone[3] (PDB-ID 6C9M), in the distal position its TPR1 and TPR2 (helices α1 to α4) rotate towards the 80S surface with shifts up to 8 Å (superposition on the rigid part of the scaffold). **(b)** In comparison to the NatA-HypK[3] complex (PDB-ID 6C95), the N-terminal helices shift by up to 5 Å in the opposite direction.

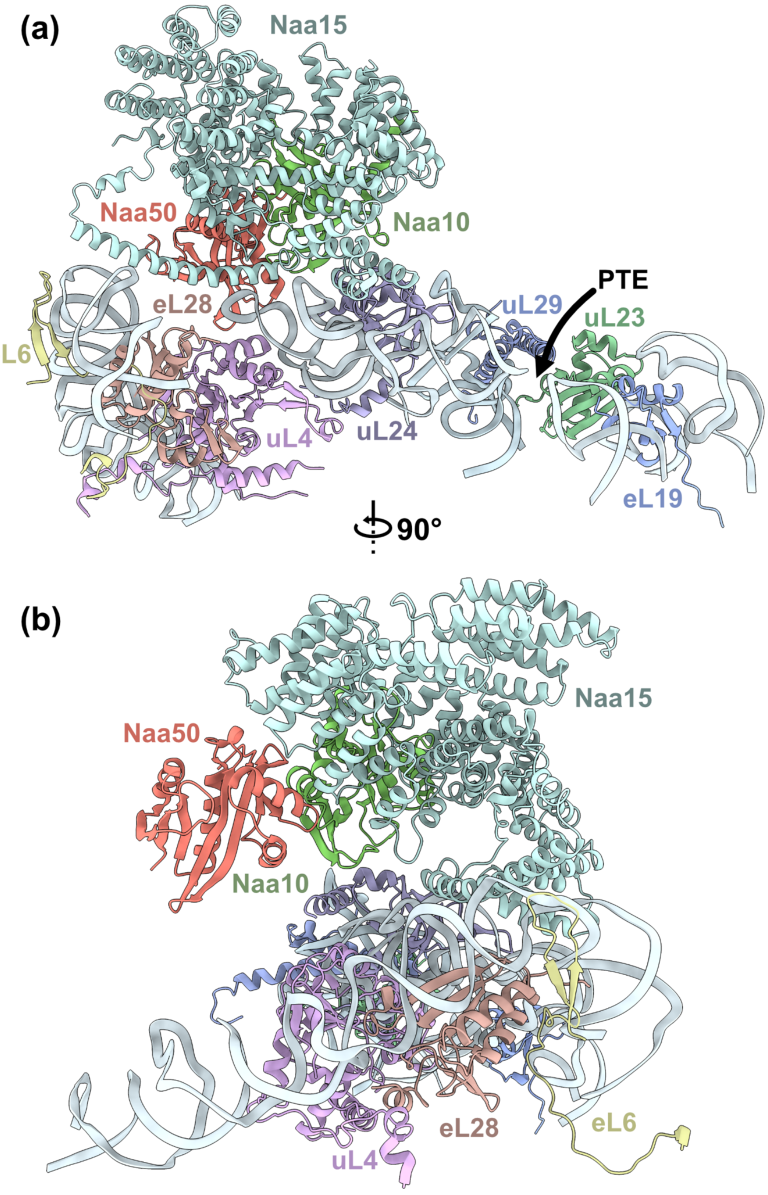

Supplementary Figure 8 | Model of human NatE ribosome binding. The cryo-EM structure of human NatE (comprising Naa10, Naa15, Naa50 (ref. [4])) superimposed on human NatA (not shown) at the distal position. **(a)** Side view along the ribosomal surface on NatE modelled at the distal site. MAP2 is not shown and the position of the PTE is indicated by an arrow. Naa50 (red) would be positioned behind Naa15 and faces away from the PTE (PDB-ID 6PPL was superimposed onto Naa15 at the distal site)[4]. **(b)** Top view onto the ribosomal surface. At the distal position, Naa50 would not contact the ribosomal surface.

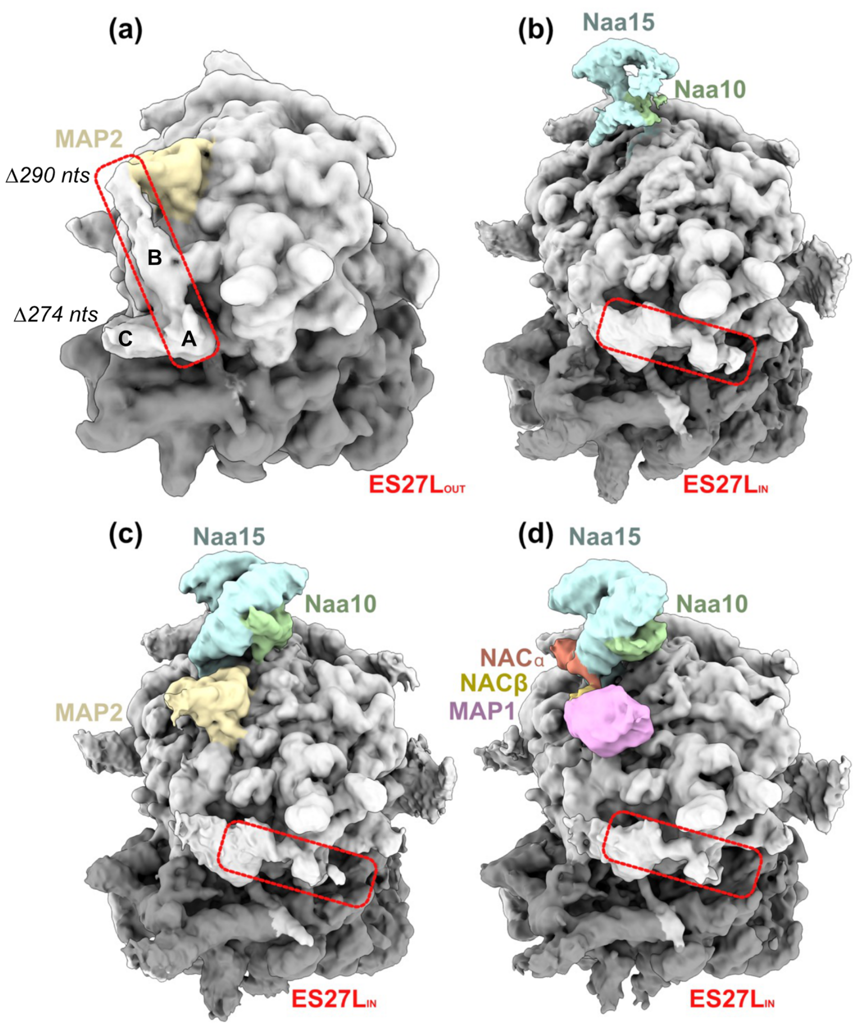

Supplementary Figure 9 | ES27L is not recruited to the $ES27_{OUT}$ position on NatA decorated ribosomes (a) In complex with MAP2 (ref. 5), ES27L is recruited to the $ES27L_{OUT}$ position on a subset of particles (Note: only minor parts of all ES27L arms (A, B, C) can be structurally traced in general, missing parts of the B- and C-arms are indicated in italics). In complex with **(b)** NatA, **(c)** NatA-MAP2 and **(d)** NatA-NAC-MAP1, ES27L is placed in the $ES27L_{in}$ position in the 60S/40S inter-subunit space.

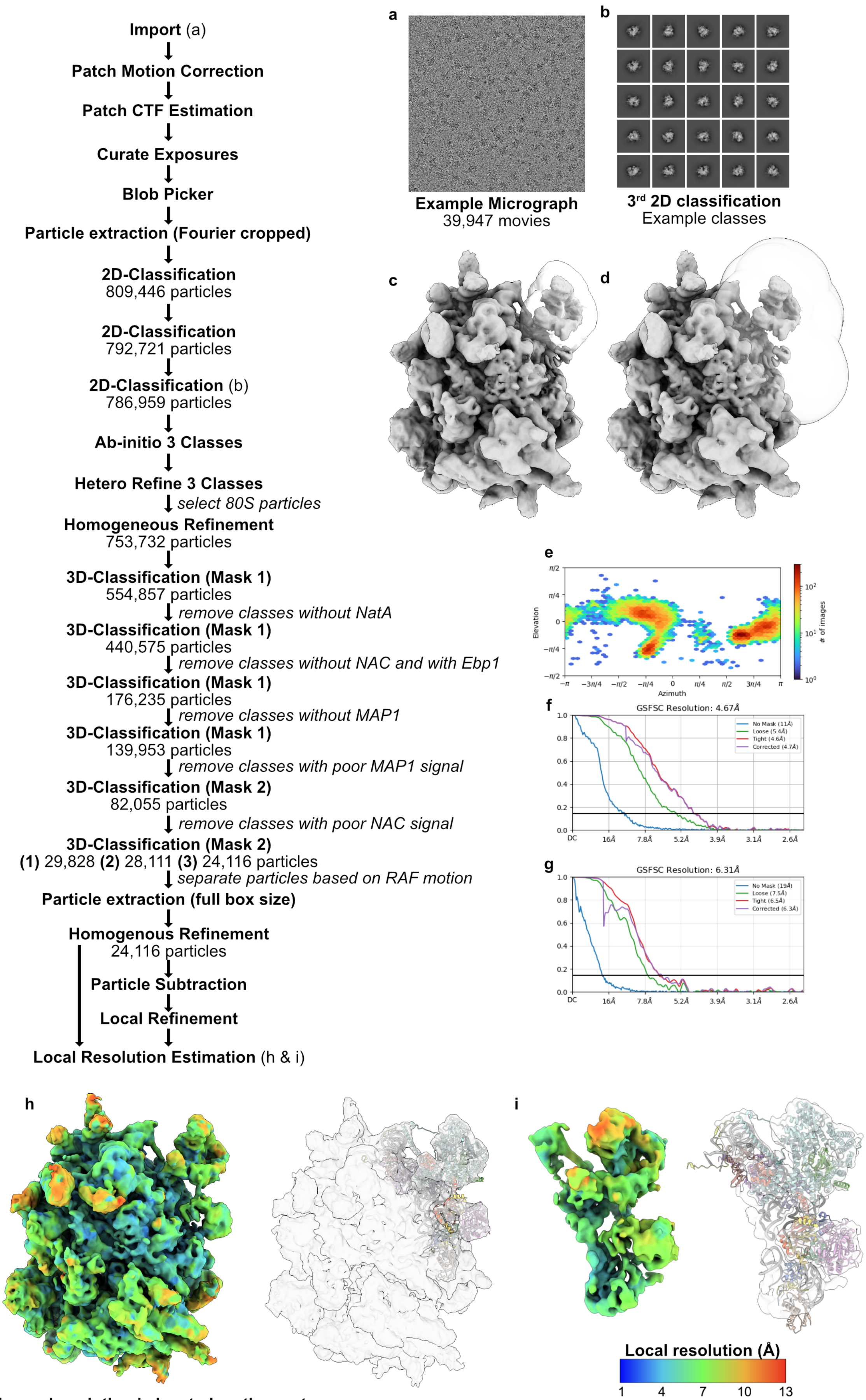

Figure description is located on the next page

Supplementary Figure 10 | Cryo-EM data processing for the NatA-NAC-MAP1-80S sample. (a) Three datasets were acquired to obtain 39,947 movies. **(b)** After pre-processing in CryoSPARC, extracted particles were subjected to three rounds of 2D-classification. Three ab-initio classes were generated from the remaining particles and used to seed a Heterogeneous refinement. 80S particles were selected and subjected to Homogeneous Refinement. **(c)** A mask (Mask 1) was generated to encompass the distal site of NatA and used to initialize a 3D classification to remove particles without NatA. The same mask was used for three additional subsequent 3D classifications to remove particles with Ebp1, without NAC, without MAP1 or with poor MAP1 signal. **(d)** A second extended mask (Mask 2) was made to encompass the binding site of NatA, NAC and MAP1 and used to initialize a fourth 3D classification to remove particles with poor NAC signal. The same mask was finally used in a fifth 3D classification into three classes to separate particles based on the motion of NatA, NAC and MAP1. One of the classes revealed the complex in a more stabilized state. Corresponding particles were re-extracted at full box size without Fourier cropping and subjected to Homogeneous refinement. **(e)** Angular distribution plot of the final Homogeneous refinement. **(f)** FSC plots of the final Homogeneous refinement. **(g)** Finally, as mask was generated to subtract the 80S signal and the mask shown in (d) was used to initialize a local refinement around MAP1, NAC and NatA. The corresponding FSC curve is shown. **(h)** Local resolution estimation of the final Homogeneous refinement. **(i)** Local resolution estimation of the final local refinement. The final models are shown next to the local resolution images within the transparent map.

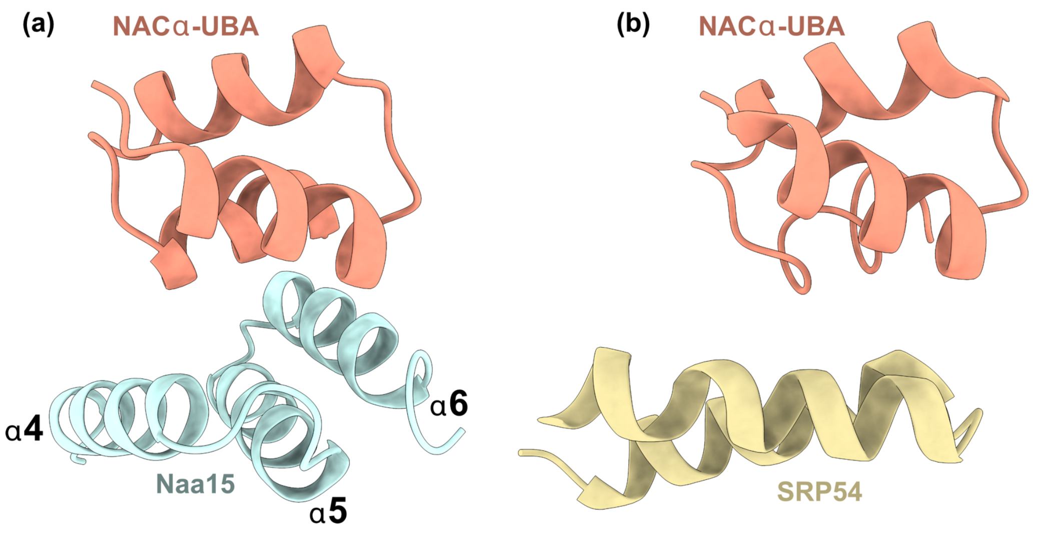

Supplementary Figure 11 | The NACα-UBA domain contacts Naa15 or SRP54. (a) NACα-UBA interacts with Naa15 helices α4 to α6. **(b)** A similar interaction between NACα-UBA (same view) is observed with the SRP54 N-domain (PDB-ID 7QWQ)[6]. Note: Helical bundles of Naa15 and SRP54 are interacting with NACα-UBA at different angles (almost 90° along the vertical axis).

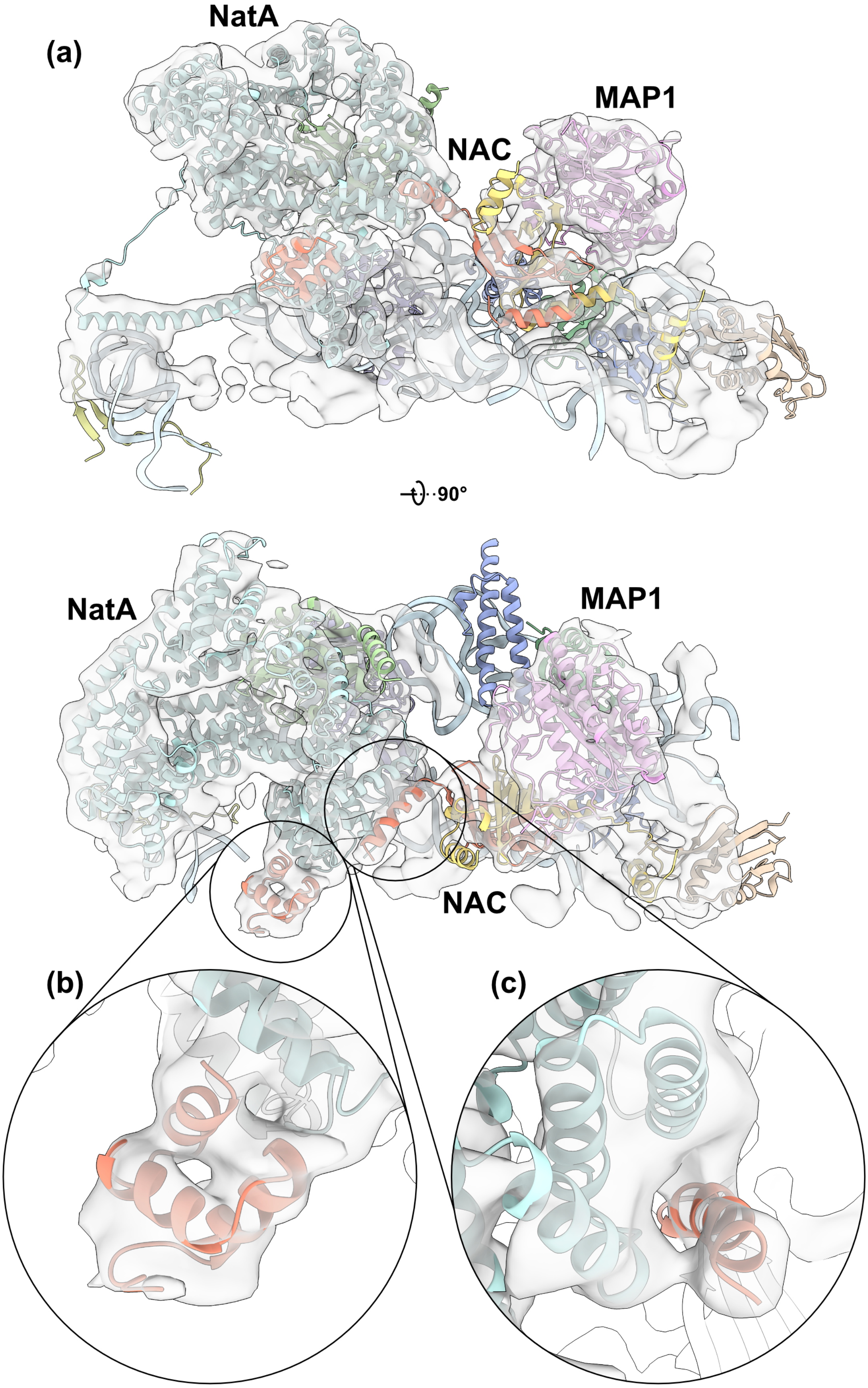

Figure description is located on the next page

Supplementary Figure 12 | Structure of NatA-NAC-MAP1 on the human ribosome. (a) Side and top view are shown onto the quaternary assembly as in Figure 1b. The cryo-EM map is shown in transparent. **(b)** The UBA domain of NAC touches down on the surface of Naa15 with a local resolution of ~8 Å **(Supplementary Fig. 10)**. **(c)** The NACα-cH interacts with the surface of Naa15 with a local resolution of 7 Å.

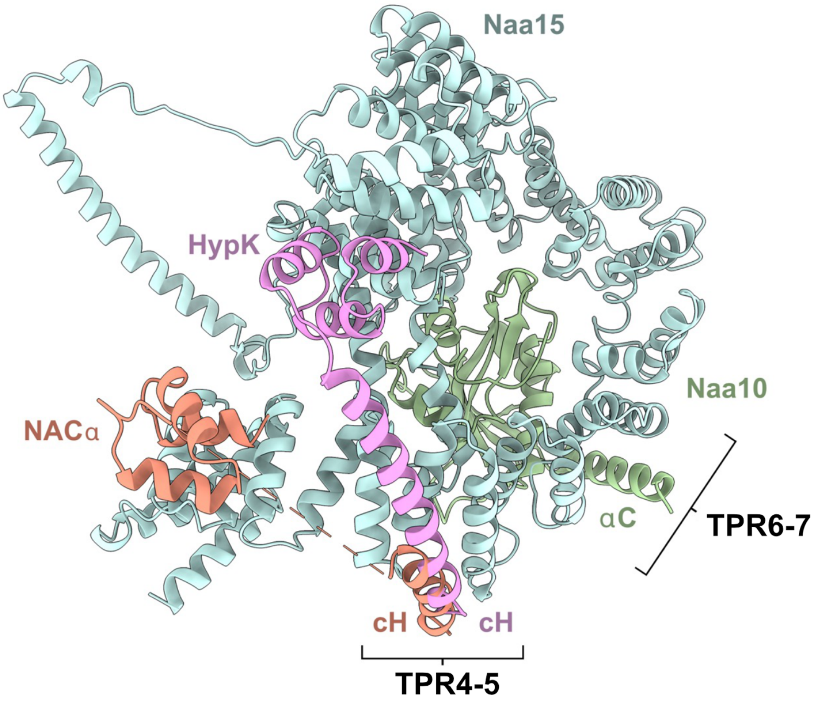

Supplementary Figure 13 |The Naa15 TPR-scaffold engages in contacts with Naa10-αC, NACα-cH and HypK-cH. The C-terminal helix of Naa10 (αC) runs along the surface of Naa15 TPR6 and TPR7 (helices α12 to α14). This parallel placement of the Naa10 C-terminal helix resembles the interaction of the NACα-cH and HypK-cH[3] with Naa15 TPR4 and TPR5 (helices α8 to α10).

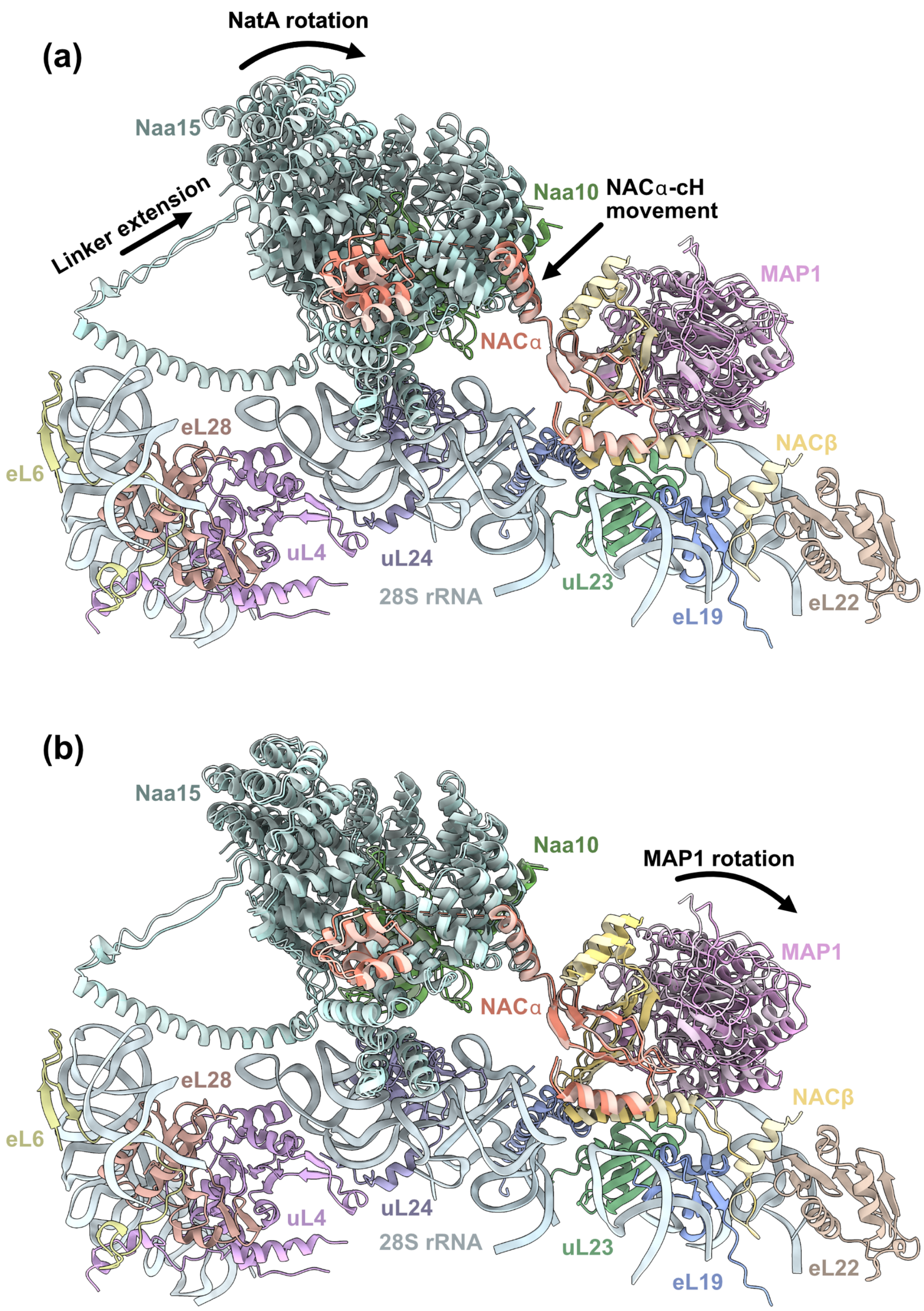

Supplementary Figure 14 | Dynamics of the quaternary NatA-NAC-MAP1-80S assembly. In the final 3D classification, particles were separated into three classes (Classes 1-3) with different orientations of NatA, NAC and MAP1. **(a)** Comparison of Class 1 (desaturated colour) with Class 2 (saturated colour). In Class 2, NatA is rotated further down towards the PTE. The linker that connects NatA to the α34 anchor is extended and the NACα-cH and UBA domain are shifted. **(b)** Comparison of Class 1 (desaturated colour) with Class 3 (saturated colour). In Class 3, the interaction between NAC and MAP1 is weaker and MAP1 is located further away from the PTE. The NAC dimerization domain is also slightly shifted.

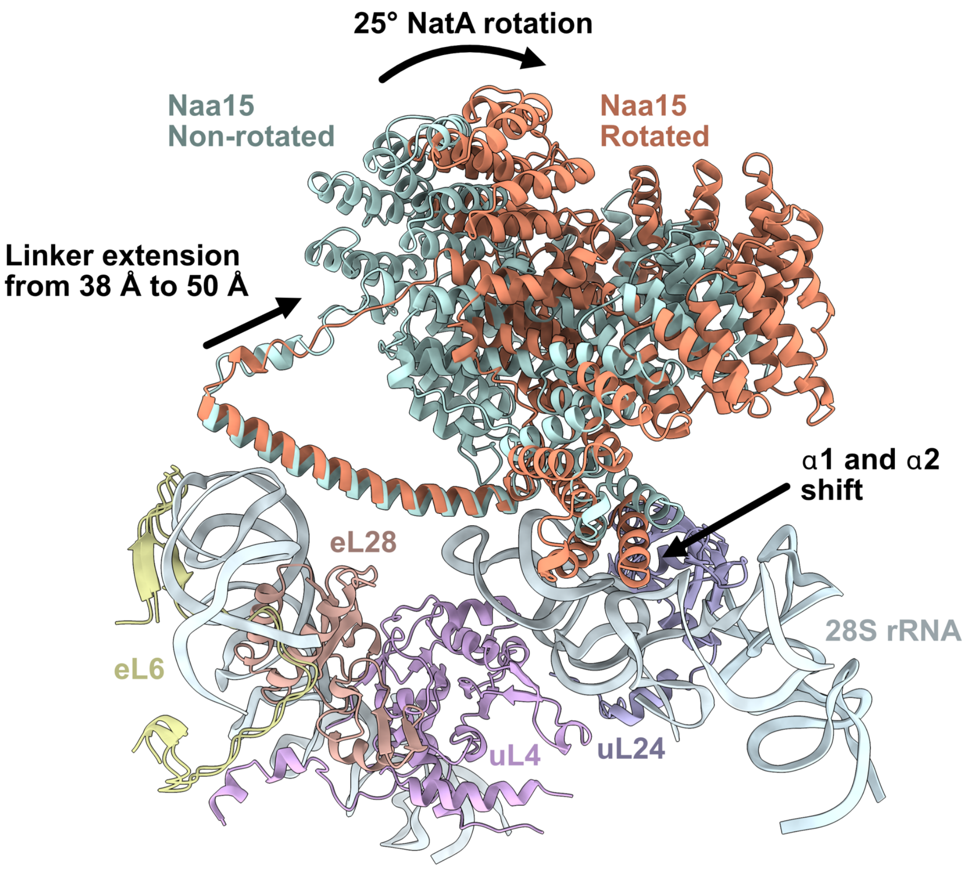

Supplementary Figure 15 | Adaptation of NatA binding in the ternary NatA-MAP2-80S complex compared to the quaternary NatA-NAC-MAP1-80S complex. In complex with NAC and MAP1, the NatA complex is rotated further down towards the PTE (red). In this rotation Naa15 overall remains anchored in place. The contacts of Naa15 helices α1 and α2 are shifted further away from uL24 in the quaternary assembly. Throughout the rotation, the flexible linker of exposed helix α34 to the TPR-scaffold stretches between 38 Å and 50 Å to enable this dynamic rotation of NatA by 25° (fulcrum around rRNA helix H24 tip).

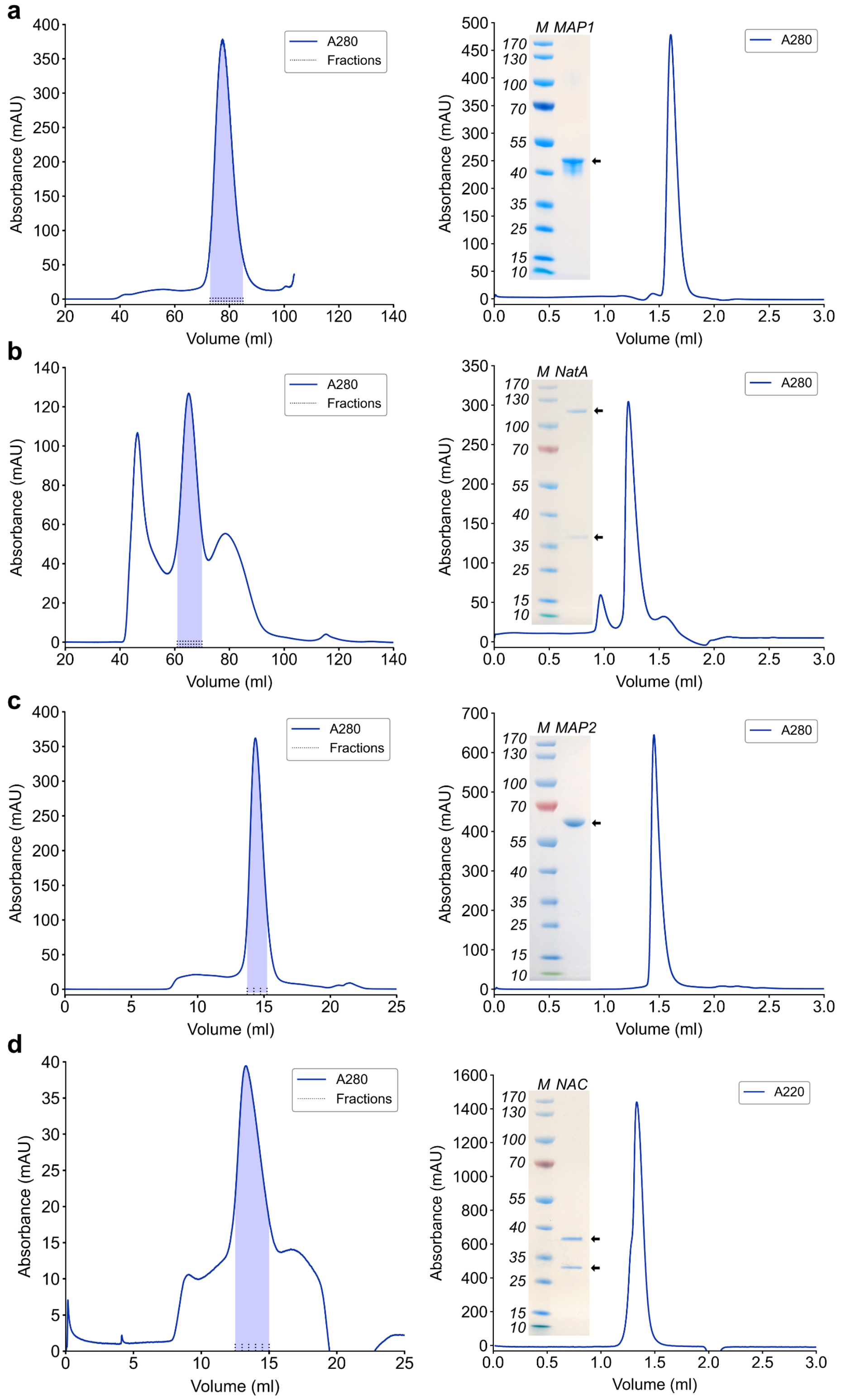

Supplementary Figure 16 | Protein purifications of ribosome associated factors. Pooled fractions from preparative size exclusion chromatography (SEC, fractions shaded in blue) were subjected to SDS-PAGE and coomassie staining, as well as analytical SEC. Preparative SEC was performed on an S200 16/600 column (Cytiva) for **(a)** MAP1 and **(b)** NatA and on an S200 Increase 10/300 GL column (Cytiva) for **(c)** MAP2 and **(d)** NAC. Analytical SEC was done on an S200 Increase 3.2/300 column (Cytiva).

Supplementary Table 1 | Cryo-EM data refinement statistics

**Model**	***Hs*NatA-*Hs*MAP2-80S**	***Hs*NatA-*Hs*NAC-*Hs*MAP1-80S**
Data collection statistics		
Microscope	Titan Krios	Glacios
Camera	K3	Falcon 3
Voltage (kV)	300	200
Magnification	105,000	120,000
Total dose ($e^-/Å^2$)	41.28 $e^-/Å^2$	53.50, 53.97 and 53.96 $e^-/Å^2$
Defocus range (μm)	-1.3 to -2.3	-0.7 to -1,7
Calibrated pixel size (Å)	0.84	1.223
Refinement statistics		
Refined particles	25,404	24,116
Resolution (Å)	2.69	4.67
Chains	15	18
Atoms	29394	31688 (Hydrogens: 0)
Residues	Protein: 2666 Nucleotide: 358	Protein: 2963 Nucleotide: 356
Water	0	0
Ligands	IHP: 1<br>CO: 2	IHP: 1
Bonds (RMSD*)		
Length (Å) (# > 4σ)	0.003 (0)	0.005 (0)
Angles (°) (# > 4 σ)	0.740 (24)	1.109 (65)
MolProbity score	1.98	2.56
Clash score	14.75	30.73
Ramachandran plot (%)		
Outliers	0.08	0.58
Allowed	3.14	4.30
Favored	96.78	95.12
Rama-Z (Ramachandran Plot, Z-score, RMSD*)		
whole (N = 951)	0.42 (0.16)	-0.03 (0.15)
helix (N = 379)	0.60 (0.14)	0.37 (0.14)
sheet (N = 107)	-0.02 (0.38)	-0.49 (0.31)
loop (N = 465)	0.12 (0.19)	-0.17 (0.18)
Rotamer outliers (%)	1.36	2.11
Cβ outliers (%)	NA	0.07
Peptide plane (%)		
Cis proline/general	0.0/0.0	1.7/0.0
Twisted proline/general	0.0/0.0	0.8/0.0
CaBLAM outliers (%)	1.45	1.76
ADP (B-factors)		
Iso/Aniso (#)	29394/0	31688/0
min/max/mean		
Protein	14.93/495.28/134.23	-0.00/990.92/204.79
Nucleotide	66.29/489.52/152.28	2.28/798.14/216.25
Ligand	115.67/250.44/122.56	235.80/235.80/235.80
Occupancy (%)		
Mean	1.00	1.00
occ = 1 (%)	100.00	100.00
0 < occ < 1 (%)	0.00	0.00
occ > 1 (%)	0.00	0.00
Model vs. Data (CC mask)	0.80	0.53
Resolution according to model vs. map FSC = 0.143 (masked) (Å)	2.9	4.8

* RMSD: root-mean-squared-deviation

Supplementary Table 2 | Oligonucleotides used for cloning

**Oligo name**	**Sequence**	**Application**
Hsmap1_fwd	CCAGGCAGTGGTAGCATGGCGG CCGTGGAG	Cloning of *pFastBacDuet-10HIS-GSGS-3C-GSGS-Hsmap1*
Hsmap1_rev	CGGGTGGCTCCAGGATCCTTAA AATTGAGACATGAAGTGAGGC	Cloning of *pFastBacDuet-10HIS-GSGS-3C-GSGS-Hsmap1*
Hsmap2_fwd	CCAGGCAGTGGTAGCATGGCGG GTGTGGAGG	Cloning of *pFastBacDuet-10HIS-GSGS-3C-GSGS-Hsmap2*
Hsmap2_rev	CGGGTGGCTCCAGGATCCTTAAT AGTCATCTCCTCTGCTG	Cloning of *pFastBacDuet-10HIS-GSGS-3C-GSGS-Hsmap2*
HsNaa15_fwd	CCCCCATCTCCCGGTACCTCAAA TTTCATTGGCCAGTTCTTCAGC	Cloning of *pFastBacDuet-naa15_naa10-GSGS-3C-GSGS-strep-II*
HsNaa15_rev	CAACTCCTAAAAAACCGCCAACA TGCCGGCCGTGAGCCTCC	Cloning of *pFastBacDuet-Hsnaa15_Hsnaa10-GSGS-3C-GSGS-strep-II*
pFastBacDuet_p10_fwd	GTTGGCGGTTTTTTAGGAGTTGT CGAG	Cloning of *pFastBacDuet-10HIS-GSGS-3C-GSGS-Hsmap1, pFastBacDuet-10HIS-GSGS-3C-GSGS-Hsmap2,* Cloning of *pFastBacDuet-Hsnaa15_Hsnaa10-GSGS-3C-GSGS-strep-II* and *pFastBacDuet-Hsnacβ_Hsnacα-GSGS-3C-GSGS-strep-II*
pFastBacDuet_p10_rev	GGTACCGGGAGATGGGGGAG	Cloning of *pFastBacDuet-10HIS-GSGS-3C-GSGS-Hsmap1, pFastBacDuet-10HIS-GSGS-3C-GSGS-Hsmap2,* Cloning of *pFastBacDuet-Hsnaa15_Hsnaa10-GSGS-3C-GSGS-strep-II* and *pFastBacDuet-Hsnacβ_Hsnacα-GSGS-3C-GSGS-strep-II*
pFastBacDuet_ph_fwd	TAAGGATCCTGGAGCCAC	Cloning of *pFastBacDuet-Hsnacβ_Hsnacα-GSGS-3C-GSGS-strep-II*
pFastBacDuet_ph_rev	CATGCTACCACTGCCTG	Cloning of *pFastBacDuet-Hsnacβ_Hsnacα-GSGS-3C-GSGS-strep-II*
pFastBacDuet_ph_fwd2	GTTGGCGGTTTTTTAGGAGTTGT C	Cloning of *pFastBacDuet-Hsnaa15_Hsnaa10-GSGS-3C-GSGS-strep-II*
pFastBacDuet_ph_rev2	GGTACCGGGAGATGGGGG	Cloning of *pFastBacDuet-Hsnaa15_Hsnaa10-GSGS-3C-GSGS-strep-II*

Supplementary References

1 Gamerdinger, M. *et al.* NAC controls cotranslational N-terminal methionine excision in eukaryotes. *Science* **380**, 1238-1243 (2023). https://doi.org/10.1126/science.adg3297

2 Abramson, J. *et al.* Accurate structure prediction of biomolecular interactions with AlphaFold 3. *Nature* **630**, 493-500 (2024). https://doi.org/10.1038/s41586-024-07487-w

3 Gottlieb, L. & Marmorstein, R. Structure of Human NatA and Its Regulation by the Huntingtin Interacting Protein HYPK. *Structure* **26**, 925-935 (2018). https://doi.org/10.1016/j.str.2018.04.003

4 Deng, S., McTiernan, N., Wei, X., Arnesen, T. & Marmorstein, R. Molecular basis for N-terminal acetylation by human NatE and its modulation by HYPK. *Nat Commun* **11**, 818 (2020). https://doi.org/10.1038/s41467-020-14584-7

5 Klein, M. A., Wild, K., Kišonaitė, M. & Sinning, I. Methionine aminopeptidase 2 and its autoproteolysis product have different binding sites on the ribosome. *Nat Commun.* **15**, 716 (2024). https://doi.org/10.1038/s41467-024-44862-7

6 Jomaa, A. *et al.* Mechanism of signal sequence handover from NAC to SRP on ribosomes during ER-protein targeting. *Science* **375**, 839-844 (2022). https://doi.org/10.1126/science.abl6459

Uncropped images of SDS-gels shown in Supplementary Fig. 16

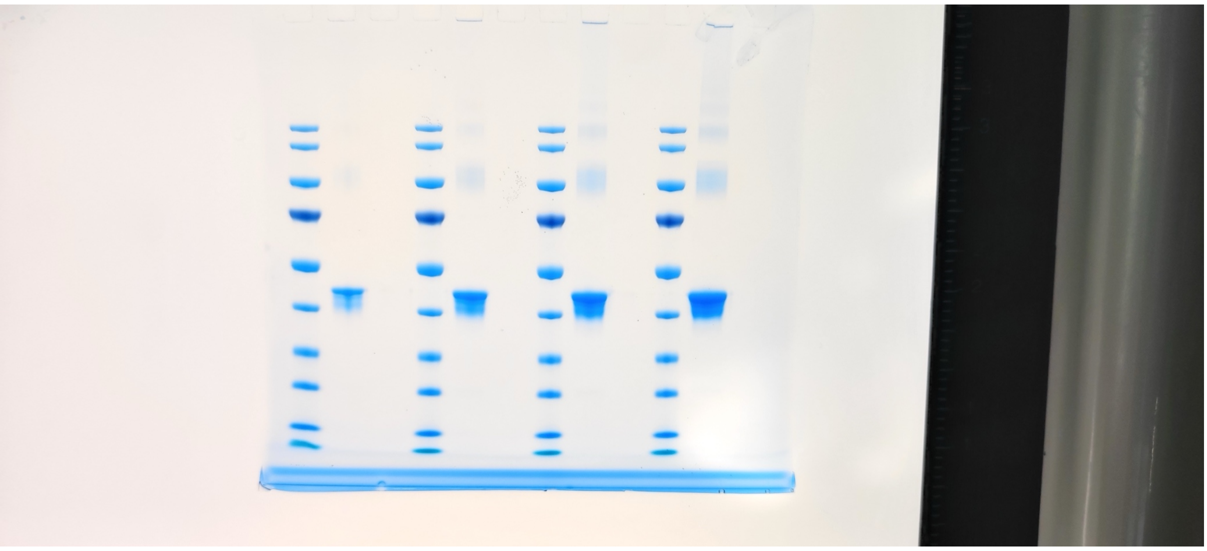

Uncropped gel shown in Supplementary Fig. 16a

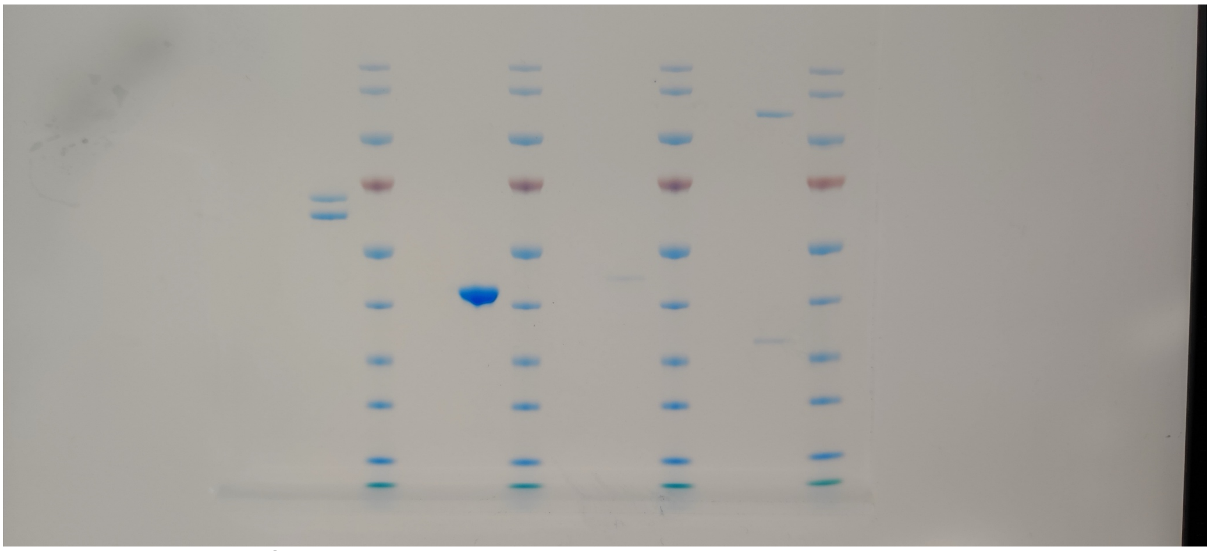

Uncropped gel shown in Supplementary Fig. 16b

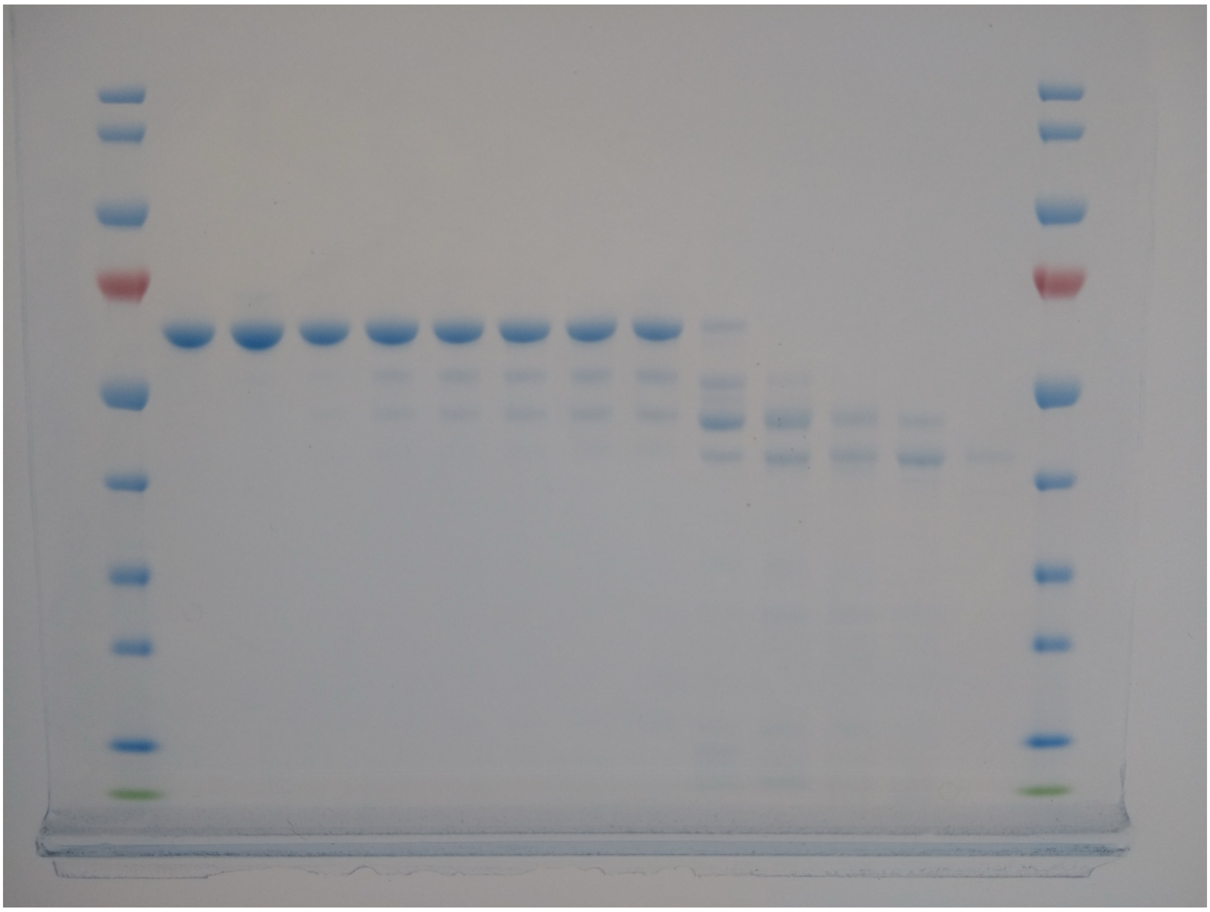

Uncropped gel shown in Supplementary Fig. 16c

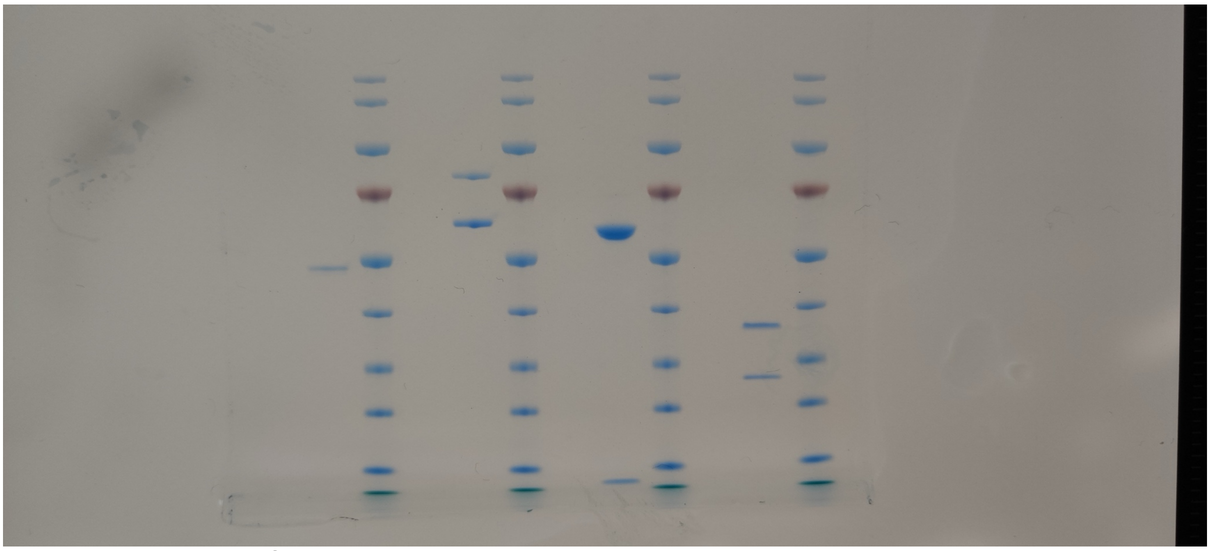

Uncropped gel shown in Supplementary Fig. 16d
